# Microbial phenomics information extractor (MicroPIE): a natural language processing tool for the automated acquisition of prokaryotic phenotypic characters from text sources

**DOI:** 10.1186/s12859-016-1396-8

**Published:** 2016-12-13

**Authors:** Jin Mao, Lisa R. Moore, Carrine E. Blank, Elvis Hsin-Hui Wu, Marcia Ackerman, Sonali Ranade, Hong Cui

**Affiliations:** 1 School of Information, University of Arizona, Tucson, 85721 AZ USA; 2 Department of Biological Sciences, University of Southern Maine, Portland, 04103 ME USA; 3 Department of Geosciences, University of Montana, Missoula, 59812 MT USA

**Keywords:** Information extraction, Phenotypic data extraction, Prokaryotic taxonomic descriptions, Microbial phenotypes, Character matrices, Support vector machine, Machine learning, Text mining, Algorithm evaluation, Natural language processing

## Abstract

**Background:**

The large-scale analysis of phenomic data (i.e., full phenotypic traits of an organism, such as shape, metabolic substrates, and growth conditions) in microbial bioinformatics has been hampered by the lack of tools to rapidly and accurately extract phenotypic data from existing legacy text in the field of microbiology. To quickly obtain knowledge on the distribution and evolution of microbial traits, an information extraction system needed to be developed to extract phenotypic characters from large numbers of taxonomic descriptions so they can be used as input to existing phylogenetic analysis software packages.

**Results:**

We report the development and evaluation of Microbial Phenomics Information Extractor (MicroPIE, version 0.1.0). MicroPIE is a natural language processing application that uses a robust supervised classification algorithm (Support Vector Machine) to identify characters from sentences in prokaryotic taxonomic descriptions, followed by a combination of algorithms applying linguistic rules with groups of known terms to extract characters as well as character states. The input to MicroPIE is a set of taxonomic descriptions (clean text). The output is a taxon-by-character matrix—with taxa in the rows and a set of 42 pre-defined characters (e.g., optimum growth temperature) in the columns. The performance of MicroPIE was evaluated against a gold standard matrix and another student-made matrix. Results show that, compared to the gold standard, MicroPIE extracted 21 characters (50%) with a Relaxed F1 score > 0.80 and 16 characters (38%) with Relaxed F1 scores ranging between 0.50 and 0.80. Inclusion of a character prediction component (SVM) improved the overall performance of MicroPIE, notably the precision. Evaluated against the same gold standard, MicroPIE performed significantly better than the undergraduate students.

**Conclusion:**

MicroPIE is a promising new tool for the rapid and efficient extraction of phenotypic character information from prokaryotic taxonomic descriptions. However, further development, including incorporation of ontologies, will be necessary to improve the performance of the extraction for some character types.

## Background

In the biological sciences, describing species has long been a basic and essential component to studies of biodiversity, systematics, and evolution. Scientists have published detailed descriptions of organisms for centuries, amassing a rich legacy of taxonomic literature that includes descriptions of phenotypic characters (i.e., the traits of an organism, such as shape, metabolic substrates, and growth conditions) for millions of species, written in natural language. Microbial descriptions of prokaryotes, in particular, also contain rich phenotypic information relating to metabolism and environmental niche space. With the genomic revolution, molecular (sequence) data is often relatively easy to obtain, and integrating molecular and phenotypic information has proven to be an important approach to study the evolution of microbial traits on a broad scale (e.g., [1–3]). However, the majority of phenotypic information is currently locked in natural language descriptions and not readily usable for bioinformatic comparative analyses.

In order to study the evolution of phenotypic traits on a large scale, i.e., covering the phenome [4]—the full set of phenotypic characters—of an organism, character information needs to be in a standard structured format (i.e., taxon-by-character matrices, which are tables with rows for taxa and columns for characters) that can be used by existing phylogenetic comparative analysis software packages, such as Mesquite [5] and MacClade [6]. Traditionally, taxon-by-character matrices (hereafter “character matrices” for short) are created manually by experts through reading and selecting phenotypic data in published taxonomic descriptions. This process is time-consuming and tedious. Hence, most studies have focused on a small number of taxa, or a small number of characters [3, 7–11]. Clearly, more efficient means of obtaining large amounts of phenotypic information for large numbers of taxa are required in order to develop a comprehensive synthesis of the distribution and evolution of diverse prokaryotic traits in the fields of microbial systematics and evolutionary biology. Information extraction techniques from text mining can provide solutions to this problem by (semi-) automatically extracting character states (i.e., character values, referred to here as “values”) from natural language text.

Text mining has been used to find patterns from plain text within the biological and biomedical domains [12]. Examples include the extraction of text to annotate protein-protein interaction networks [13, 14], metabolic pathways [15], gene regulatory networks [16], and signaling networks [17]. Within a text mining system, information extraction is an important task to extract knowledge elements from texts. The extraction targets are typically entities (objects of interests in the domain, such as protein names), the relationships among entities (e.g. protein-protein interactions), and biological events (e.g. regulatory events) [18].

The techniques used to recognize the extraction targets may be categorized as employing 1) an existing knowledge base (e.g. term dictionaries or ontologies), 2) machine learning or computational statistics methods, and/or 3) Natural Language Processing (NLP) methods. These techniques are typically used in combination to achieve the best performance. The discovery of domain entities is often achieved using an existing knowledge base and enhanced with machine learning mechanisms. Machine learning algorithms use statistical methods to model the problem of interest and make predictions based on the model [19]. For example, Support Vector Machine (SVM) is a general-purpose, robust, supervised classifier based on mathematical theory of hyperplane arrangements [20] that can be trained with tagged examples, then used to classify new examples. Because of its robustness and wide applicability, SVM is used in text mining for document classification [21], sentence classification [22], named entity recognition [23–25], and beyond (e.g., [26, 27]). Extensively used in information extraction, NLP techniques combine language theories and statistical methods to analyze syntactic structure of plain text [28], entity roles, and their relationships. To capture a variety of phenotypic characters from microbial descriptions, we applied multiple techniques, including SVMs and syntactic analyses.

In biodiversity domains, significant strides have been made recently in the development of automated text mining and natural language processing approaches to extract morphological data from the taxonomic literature of plants and animals. One such example is CharaParser [29] developed for extracting morphological characters from semi-structured taxonomic descriptions written in a telegraphic style [30, 31]. Initial tests (not shown), however, showed that CharaParser performed poorly at extracting data from prokaryotic taxonomic descriptions because these descriptions contain little morphological data, but rather focus on physiological and ecological traits (Table 1).

**Table 1:** High-level categories (indicated by stars) and characters implemented in MicroPIE

Categories*/characters	Example source sentences
G + C Content*	
%G + C	DNA G + C content is **33.1–34.4 mol%**.
Cell Morphology*	
Cell Shape	Cells are **slender, cylindrical, sometimes crooked rods** that are 0.35–0.5 μm wide and 2.5 μm long and occur singly or in pairs, or in longer chains.
Cell Diameter	Cells are **0.3–0.5 μm** in diameter.
Cell Length	In glucose broth, the bacilli are longer, up to **4 μm** long, and often occur in pairs.
Cell Width	In addition, cells have an outer diameter of 1.5–3.0 μm and width of **0.5–0.9 μm**.
Cell Relationship & Aggregations	A few strains grow **in chains of 3 to 6 elements** in glucose broth.
Gram Stain Type	The cells are **Gram-negative**, long, slender rods or sometimes filaments, motile by gliding.
External features	Cells are frequently occurring in chains and **producing appendages** in older cultures.
Internal features	**No spores** are observed.
Motility	Cells are **non-motile**, extremely pleomorphic
Pigment Compounds	**Pigmented red** due to carotenoids.
Growth Condition*	
NaCl Minimum	Growth requires **at least 1.7 M NaCl, optimally 3.0–4.3 M NaCl**.
NaCl Optimum	
NaCl Maximum	
pH Minimum	Growth occurs at temperatures in the range **38–68 °C (optimum, 60 °C**) and at **pH 1.8–4.0 (optimum, pH 3.0**).
pH Optimum	
pH Maximum	
Temperature Minimum	
Temperature Optimum	
Temperature Maximum	
Salinity Requirement for Growth	**Requires sea salts** for growth.
Aerophilicity	**Strictly anaerobic. Obligately anaerobic.**
Magnesium Requirement for Growth	**Mg2+** and **Ca2+** enhance growth.
Vitamins and Cofactors Used For Growth	**Vitamin K1** and **haemin** are required for growth.
Antibiotic Physiology*	
Antibiotic Sensitivity	Sensitive to (μg per disc) **tetracycline** (30), **rifampicin** (25) and **vancomycin** (30), but resistant to ampicillin (25), erythromycin (15), streptomycin (10) and gentamicin (10).
Antibiotic Resistant	The type strain is resistant to **erythromycin**.
Colony Morphology*	
Colony Shape	On MA, colonies are **convex**, translucent, shiny and smooth.
Colony Margin	Colonies are golden-yellow, circular and convex, with an **entire edge** on MA.
Colony Texture	On MA, colonies are convex, **translucent, shiny and smooth**.
Colony Color	Colonies are **golden-yellow**, circular and convex, with an entire edge and a butyrous consistency on MA.
Metabolic End Products and Secondary Metabolites*	
Fermentation Products	**Acid** is produced from glucose and fructose.
Other Metabolic Products	**Acetoin** is produced.
Microbe-Host Interaction*	
Pathogenic	Pathogenic for **humans**, causing wound infections.
Disease Caused	Pathogenic for honeybees in natural and experimental **oral infections**.
Pathogen Target Organ	Nodosus is infected **hooves** of sheep and goats.
Haemolytic/Haemadsorption Properties	**No hemolysis** occurs on rabbit blood agar.
Metabolic Substrate*	
Organic Compounds Used Or Hydrolyzed	Utilize **carbohydrates** for growth.
Organic Compounds Not Used Or Not Hydrolyzed	Arabinose, mannose, N-acetylglucosamine, maltose are used as sole carbon and energy source but not **mannitol, gluconate, caprate, adipate, citrate and malate**.
Inorganic Substances Used	Does not require yeast extract for growth, and can use **inorganic nitrogen sources such as sodium nitrate and ammonium chloride**.
Inorganic Substances Not Used	Does not require **yeast extract** for growth, and can use inorganic nitrogen sources such as sodium nitrate and ammonium chloride.
Fermentation Substrates Used	Ferments **proteinaceous compounds**.
Fermentation Substrates Not Used	And no acid is produced from **glycerol, erythritol, L-arabinose, D-ribose, or potassium 5-ketogluconate**.

Example source sentences for each character within each category are provided. Bolded text in the source sentences indicates the values that MicroPIE should extract

Domain-independent information extraction systems have been reported, which include Open Information Extraction (OIE) [32] and DeepDive [33]. OIE extracts entity pairs connected through verbs so that some kinds of character values could be extracted; for example, “bacteria” will be returned as an appropriate answer for the question “what produces methane?” by OIE from the sentence “These bacteria eat through practically anything and produce methane gas in the process.” However, although OIE relies on textual context to extract information, it was not adequate for our task, which required substantial domain knowledge. DeepDive provides statistical machine learning and inference support for building domain-specific, information extraction systems for knowledge base construction from texts [33]. DeepDive has been used successfully to build information extraction systems for specific domains, for example, Pharmacogenomics [34]. However, it is essentially an interactive system and requires considerable user-developer interactions and input from domain experts.

Algorithms/programs specifically designed to extract microbial-related characters also exist. Humphreys et al. [35] designed the EMPathIE system based on domain concepts and various NLP techniques to extract enzyme and metabolic pathways. Their system was evaluated on a small corpus with seven journal articles, and the performance was less optimal (recall = 23%, precision = 43%) [36]. Czarnecki et al. [37] proposed a text-mining system for extracting three types of metabolic reactions. The task was accomplished through two steps: entity recognition by existing text mining tools (OSCAR3 [38] and BANNER [39]) and reaction extraction by scoring the permutations of the recognized entities in sentences selected via heuristic/syntactic rules. The performance for these methods was poor to high, having recall scores ranging from 20 to 82% and precision scores ranging from 40 to 88% [37]. These existing text mining systems could provide part of the solutions to phenotypic character extraction but were insufficient for extracting extensive, comprehensive microbial phenotypic traits needed to generate character matrices.

Ontologies are used not only as a source of knowledge in information extraction systems, they are also used as a way to standardize the results of information extraction. Matching natural language phrases to ontology terms is often treated as a separate step/task after the task of extracting character information from text. For example, Bacteria Biotope Task of the BioNLP Shared Task [40] includes a task of extracting bacteria and their habitat information, and a separate task of matching extracted entities to an ontology.

**Fig. 1:**
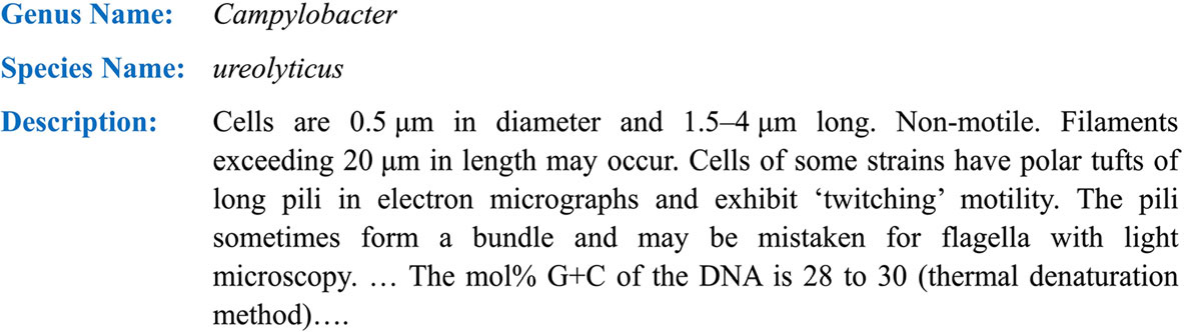
An example input description to MicroPIE, simplified from [61], used with permission

**Fig. 2:**
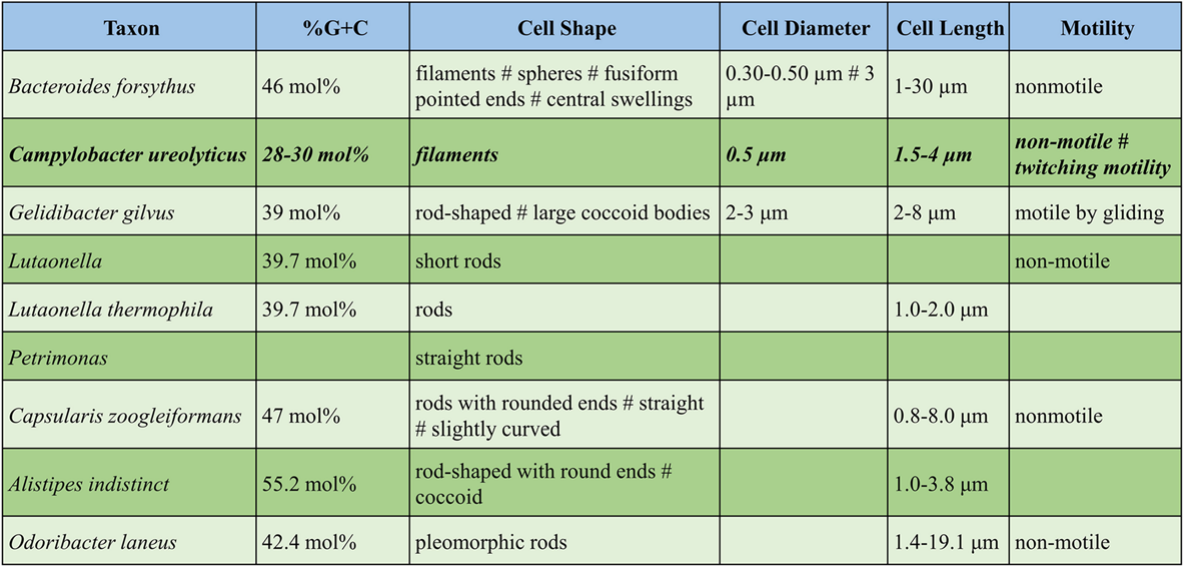
Part of a hypothetical output matrix. The row in *bold* corresponds to the description in Fig. 1

This paper reports the development and evaluation of a software application, MicroPIE (Microbial Phenomics Information Extractor) which extracts 42 microbial phenotypic characters from prokaryote descriptions and generates a character matrix. Examples of characters (and their values) include Cell Diameter (0.5 μm), Cell Length (1.5–4 μm), Motility (non-motile), and G + C Content (28–30 mol%). The input to MicroPIE is a set of prokaryotic taxonomic descriptions (natural language text; e.g. Fig. 1). An example output matrix is shown in Fig. 2. In this paper we focus on character extraction from natural language descriptions. The standardization of the extracted characters using ontologies to facilitate data sharing and integration (e.g. [41]) is discussed in future work.

Here, we describe the process of defining the extraction targets for MicroPIE, its system architecture and character extraction methods, as well as its performance evaluation metrics. We then report the performance results of MicroPIE with and without its character prediction component, and compare its performance to the performance of a group of undergraduate microbiology students. After discussions on system performance and algorithm refinements, we conclude the paper with a future development plan for MicroPIE.

## Methods

### Extraction target identification and selection

Exploratory studies were first conducted to identify the characters that needed to be extracted. To broadly represent the diversity of text and traits in prokaryotic taxonomic descriptions, a corpus of 625 descriptions was initially sampled from three evolutionarily distant groups (Cyanobacteria, 98 descriptions; Archaea, 422; and Mollicutes, 105). Published taxonomic descriptions were obtained as PDF files from a variety of journals, including International Journal of Systematic and Evolutionary Microbiology [42], Proceedings of the National Academy of Sciences of the United States of America [43], etc. Each taxonomic description was semi-automatically transferred into a text file. PDF-to-text conversion and/or formatting errors were manually corrected so that the extracted text matched the original.

The collected microbial taxonomic descriptions were then segmented to 8536 sentences using the Stanford CoreNLP Toolkit [44]. Two R packages implementing LSA (Latent Semantic Analysis) [45] and topic models [46] were used to analyze the content of microbial taxonomic descriptions. This analysis identified 72 topics as raw categories, such as G + C content, growth temperature, and cell size. These topics were then combined, resulting in a set of 8 high-level categories that cover general traits of prokaryotes. Consulting the taxonomic description corpus, the characters were specified under each category and a set of 42 characters were defined as the extraction targets for MicroPIE (Table 1).

### MicroPIE system architecture

**Fig. 3:**
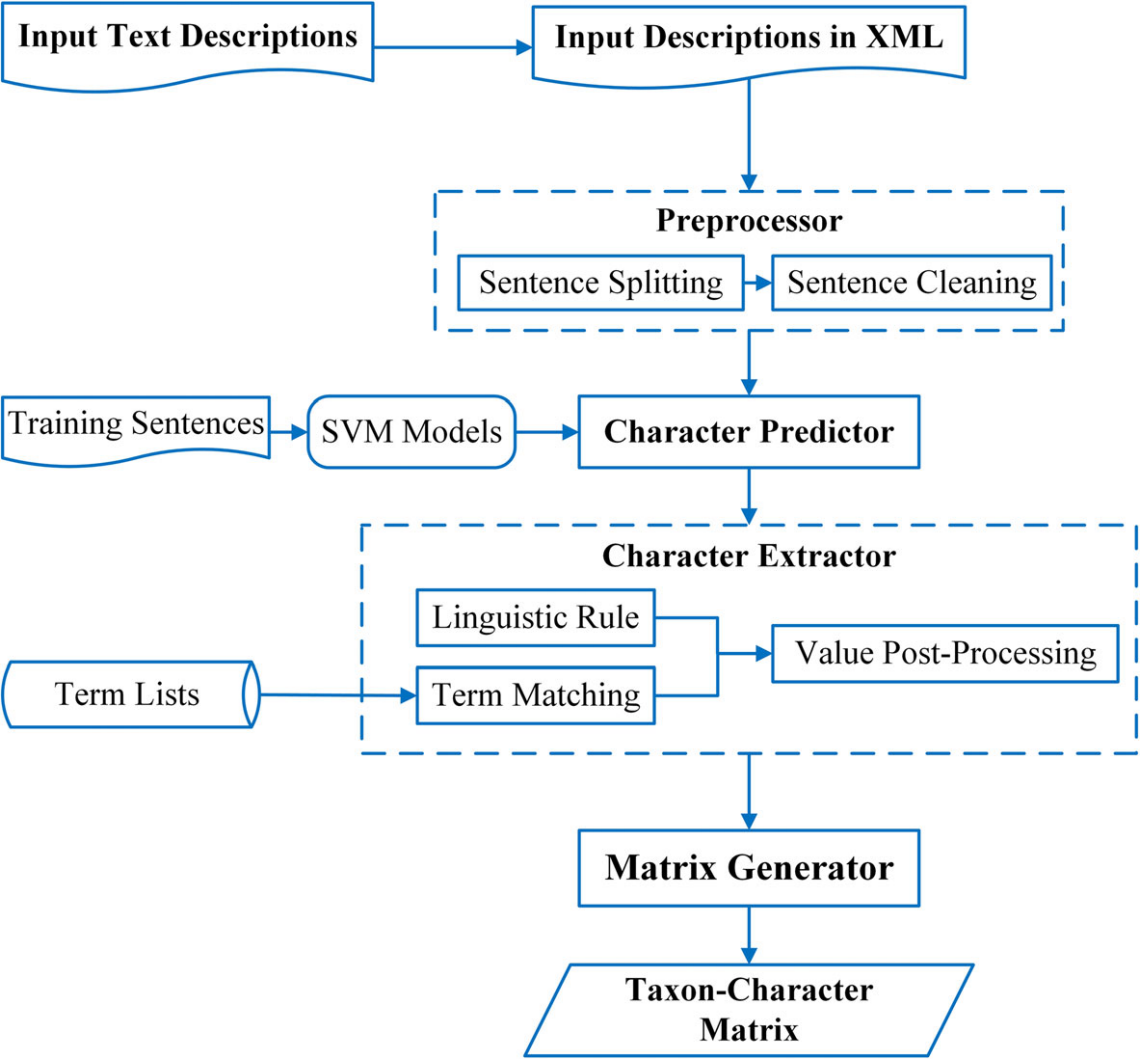
System architecture of MicroPIE

Figure 3 shows the system architecture of MicroPIE. Text input is first converted into a simple XML format where publication metadata (author, title, and date), taxon names and description paragraphs are wrapped in separate elements. Next, the XML files are analyzed by MicroPIE Preprocessor, Character Predictor, Character Extractor, and Matrix Generator in sequence to produce a taxon-by-character matrix. MicroPIE does not automatically detect/remove descriptions that are potentially repetitive or highly similar, because they could represent different taxon concepts [47], the subject of research for some potential users of MicroPIE.

The MicroPIE Preprocessor component has two main steps: Sentence Splitting and Sentence Cleaning. In the Sentence Splitting step, the description paragraphs are split into sentences using Stanford CoreNLP [44]. In the Sentence Cleaning step, sentences are normalized by replacing predefined XML entities (e.g., “&lt;” is replaced by “<”) and characters that are not compatible with the UTF-8 charset, and by removing non-alphanumeric characters (e.g., “⇑”). MicroPIE Preprocessor supplies the subsequent components with clean, normalized sentences to reduce potential syntactic parsing errors.

The Character Predictor consists of a set of trained SVM classifiers (LibSVM [48]), one for each character. The function of the Character Predictor is to predict the characters a sentence describes by classifying the sentence into one or more of the 42 character classes (each character in Table 1 is a class). The SVM classifier had been trained with the 8481 sentences from the 625 descriptions mentioned before, then semi-manually labelled with appropriate characters (classes). For characters with fewer than ten training sentences, more sentences were added by searching Google Scholar. In total, 17,147 training instances (one training instance = one sentence + one character label) were generated. The number of training sentences per character ranged from tens to more than 1000. When training the SVMs for a character, training sentences labeled with the character were used as its positive training examples, and the same number of negative training examples were randomly selected from the remaining sentences. This is done to keep the positive and negative training examples balanced as SVMs are sensitive to unbalanced training examples [49]. The trained SVMs achieved an average 98.6% precision and 99.5% recall in character prediction as evaluated via a 10-fold cross validation.

The Character Predictor uses each of the 42 trained LibSVM classifiers to classify a sentence. For example, the sentence, “Cells are 0.3–0.5 μm wide and 8.0–15.0 μm long”, may be classified to both Cell Width and Cell Length characters. After character prediction, sentences are passed to the appropriate Character Extractors to extract the target values.

**Fig. 4:**
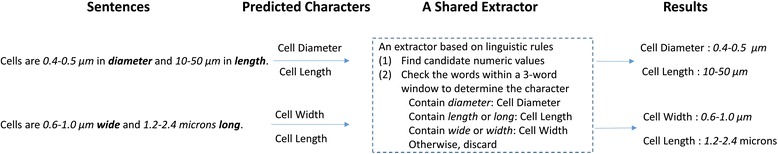
A shared extractor based on linguistic rules for the characters Cell Diameter, Cell Width, and Cell Length

A variety of character extractor algorithms have been implemented in the Character Extractor component; and they can be categorized into two main groups: linguistic rules and term matching (details in the section below). Since one sentence may be classified into multiple characters by the Character Predictor component, the sentences may be processed by multiple extractors. On the other hand, one extractor may be used for several character classes of similar nature. For example, Cell Diameter, Cell Width, and Cell Length are processed by the same extractor that is based on linguistic rules shown in Fig. 4. The target values for the three characters share a similar linguistic pattern: the keywords that indicate the character follow the candidate numeric values within a 3-word window.

The results from these extractors are then post-processed using predefined heuristic rules to generate the final results. For example, the Cell Length value “1.2–2.4 microns” in Fig. 4 was standardized as “1.2–2.4 μm” in the post-processing step. The last component of MicroPIE, Matrix Generator, creates a taxon-by-character matrix with the values extracted for the characters. Multiple values for a character are separated by number signs (#).

### MicroPIE character extraction methods

#### Linguistic rules

The linguistic rules for a character were identified from its SVM training examples when developing MicroPIE. Text segments are extracted as candidate character values if the text segments satisfy relevant linguistic rules. Linguistic rules used in MicroPIE include regular expressions, Part-Of-Speech (POS) tag patterns and syntactic patterns [50]. In some cases, multiple linguistic rules are often integrated in one character extractor to deal with varied textual expressions of a character.

Regular expressions are used to extract character values with clear lexical clues; for example, a minimum and maximum for the range of pH values (i.e., 6–8) at which a bacteria could grow can be extracted from “Grows at pH 6–8”. Regular expressions only exploit lexical patterns of the sentences. POS tag patterns, in contrast, also take the POS tags of the words into consideration. POS tag patterns often consist of POS tags and specific words. For example, one simple pattern “<CD > mol%” is used to extract the number with the POS tag “CD” (i.e., a cardinal number) followed by “mol%” as the value of G + C Content.

**Fig. 5:**
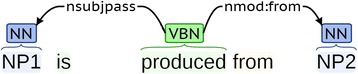
An example of a syntactic pattern used in MicroPIE

Syntactic patterns used in MicroPIE are the character-indicating syntactic dependencies [44] identified from microbial descriptions. For example, if a sentence classified as Fermentation Substrate Used contains the phrase “is produced from”, then this verb phrase often indicates fermentation related characters (Fig. 5): Noun Phrase 1 (NP1), or the subject (nsubjpass dependency) of the verb phrase, is often a fermentation product, while Noun Phrase 2 (NP2), or the object (nmod dependency), is a fermentation substrate.

#### Term matching

The term matching approach references the knowledge base which consists of lists of terms (2563 terms) that were created manually for 29 of the 42 characters, as well as terms from other ontologies such as the Plant Ontology [51] (3136 terms) and the Human Disease Ontology [52] (27,651 terms). The knowledge base provides the domain knowledge MicroPIE cannot gather from the text descriptions alone. For example, if it is known that “chains” is associated with the character Cell Relationships & Aggregations, a value (“short chains”) may be extracted from the sentence “some cells are arranged in short chains”.

Noun phrases of sentences are first obtained by applying heuristic rules with POS tag information. Each term in the term list is used to formulate a regular expression. Then, the regular expressions for a character are applied to a noun phrase to determine whether this phrase is a candidate value of the character. For some characters whose character states appear as in the term list without extra variations, the character values are extracted by checking whether the terms exist in the sentences. In addition, syntactic rules can be combined with term matching to disambiguate the characters of the phrases through restricting the dependency roles of the matched phrases in the sentence. For example, if the NP2 in Fig. 5 matches with a term in the term list of Fermentation Substrate Used, the confidence in this phrase being the character value is further improved.

It is important to note that use of term matching alone is not sufficient for the character extraction task, because (1) these terms could occur in descriptions outside of the designated character classes, and (2) extraction targets are often more complex than a single phrase. To overcome the insufficient coverage of the term lists for some characters, coordinated noun phrases e.g., “chloramphenicol, streptomycin and kanamycin”, are identified. If one phrase in the coordinated noun phrases is recognized as a character value, all phrases in the coordination are assumed to be the values for the character.

### MicroPIE performance evaluation

The performance of MicroPIE was evaluated against a gold standard character matrix (referred to here as the gold standard matrix, or GSM) prepared by microbiologists on our team, and also compared to the performance of undergraduate microbiology students who manually extracted a subset of microbial phenotypic characters.

#### Development of the gold standard matrix

Taxonomic descriptions (133) from International Journal of Systematic and Evolutionary Microbiology [42] were used to create a gold standard matrix (GSM). The GSM contains the ground truth of the characters for each taxon. GSM creation was time-consuming due to the ambiguities in some taxonomic descriptions or the complexity of the characters and syntax. To accelerate the manual process, the trained SVM classifiers were used to predict the characters in the sentences. Any missed or incorrect predictions of the SVM classifiers were manually identified and corrected. Despite the use of the SVM classifiers, the generation of the GSM took well over 180 person-hours.

In order to compare the character values in the GSM to machine-generated values from MicroPIE, the values were formulated in a structured way. String-based values were formatted as “negation │ modifier │ main value” with the main value as the only required field. Modifiers are phrases that refine the main value in terms of degree, extent, or frequently. For example, “not strictly anaerobic” is formatted as “not │ strictly │ anaerobic”. The format for characters with numerical values had additional fields, “negation │ modifier │ main value │ unit │ sub-character”, with the main value being the required field which could hold a single or range numeric value. The field sub-character is only used to indicate the kind of salt (NaCl, sea salts, MgSO4, etc.), for the characters of NaCl Minimum, NaCl Optimum or NaCl Maximum. For example, “2–3% NaCl” is formatted as “2–3 │ % │ NaCl” (negation and modifier fields are absent in this example).

Of the 133 taxonomic descriptions in the GSM, 22 were used to optimize/test the character extractors, and the remaining 111 were used for the evaluation of the system.

#### MicroPIE performance evaluation metrics

MicroPIE performance metrics were calculated by comparing the character matrix produced by MicroPIE against the GSM. Precision (P), Recall (R), and F1-measure (F1) scores were adopted to evaluate MicroPIE performance for each character. For each value within a character, a hit score was assigned denoting how well the extracted value matches the GSM value on a scale of 0–1, with 1 being a perfect match, 0 being no match and 0.5 being a partial match. From these hit scores, P, R and F1 were calculated as follows:

$$ \mathrm{P}=\frac{\mathrm{sum}\ \mathrm{of}\ \mathrm{hit}\ \mathrm{scores}}{\#\ \mathrm{of}\ \mathrm{all}\ \mathrm{extracted}\ \mathrm{values}} $$

$$ \mathrm{R} = \frac{\mathrm{sum}\ \mathrm{of}\ \mathrm{hit}\ \mathrm{scores}}{\#\ \mathrm{of}\ \mathrm{values}\ \mathrm{in}\ \mathrm{G}\mathrm{S}\mathrm{M}} $$

$$ \mathrm{F}1=\kern0.5em \frac{2\mathrm{P}\mathrm{R}}{\mathrm{P}+\mathrm{R}} $$

There were various scenarios to consider in scoring the match between an extracted and a GSM value. Thus, we designed both a rigid method and a relaxed method to calculate hit scores which in turn produces rigid and relaxed P, R, and F1 scores. If all formatted fields of the two values matched exactly, both the rigid and the relaxed hit scores were 1 (Example 1 in Table 2). A score of 0 was given if the main value was not extracted (empty value), a wrong value was extracted, or the negations did not match (Example 2 in Table 2). If the main values and negations matched, but the other fields did not, the rigid hit score was 0.5 and the relaxed hit score was 1 (Example 3 in Table 2). If the negations matched, and main values partially matched, both the rigid and relaxed hit scores were set to 0.5 (Example 4 in Table 2). For characters with multiple values, each of the values was compared individually with the values in GSM and the sum of the individual scores was the final hit score (Example 5 in Table 2).

**Table 2:** Examples of how performance evaluation metrics were calculated

Example #	Character	GSM value	# GSM values	Extracted value	# extracted values	Rigid hit score	Relaxed hit score
1	%G + C	55.2 │ mol%	1	55.2 │ mol%	1	1	1
2	Organic Compounds NOT Used or NOT Hydrolyzed	esculin	1	Neither lactate nor pyruvate	1	0	0
3	Cell Shape	short plump │ rods	1	plump │ rods # short	2	0.5	1
4	Motility	not │ motile by gliding	1	not │ motile	1	0.5	0.5
5	Fermentation Substrates Used	arbutin # salicin # D-raffinose # D-mannose # sucrose # melibiose	6	melibiose # sucrose # D-mannose # D-raffinose # salicin # Most strains ferment arbutin	6	5.5	6
Total			10		11	7.5	8.5

Rigid and relaxed hit scores measuring the match between extracted values and gold standard matrix (GSM) values, illustrated with examples

Hit scores were computed using string matching. For imperfect matches, we carried out additional manual review using a designed user interface through Amazon Mechanical Turk [53]. This method allowed for the manual adjustment of relaxed scores, if warranted, since the automatic scoring algorithm biased the extracted values because it often assigned 0 to partial matches. It also allowed, in some cases, for the correction of GSM values that were initially coded incorrectly, and for the rescoring of these values. Additionally, we compared the sum of the relaxed hit scores to the sum of the total value occurrences in the GSM to calculate the % of values that MicroPIE extracted successfully.

#### Comparison to a student-generated character matrix

Undergraduate microbiology students at University of Southern Maine and Kenyon College created a matrix in their guided class projects for 12 of the 42 prokaryotic characters from a subset (46) of the 111 taxonomic descriptions in 2014. The relaxed evaluation scores for characters in the student-generated matrix compared to the GSM were calculated manually because the values in the student-generated matrix were formatted differently from the GSM. The statistical significance of the performance differences between the student-generated matrix and the MicroPIE output was evaluated by using Student's *t*-Test comparing description-based Relaxed_P, Relaxed_R, and Relaxed_F1 scores.

#### Impact of character predictor on MicroPIE performance

In MicroPIE, the Character Predicator triages sentences into different Character Extractors (Fig. 3). To assess the effects of the Character Predictor component on character extraction results, the same matrix generation task was run with and without Character Predictor. In the latter case, sentences were passed into Character Extractor from Preprocessor directly. The performance differences in the evaluation metrics between MicroPIE with/without Character Predictor were compared.

## Results

### The performance of MicroPIE with character predictor

It took 47 min and 45 s for MicroPIE to process the 111 taxonomic descriptions used as evaluation dataset collected from International Journal of Systematic and Evolutionary Microbiology [42] (total words: 24,334) and output a matrix, running on a standard PC laptop (OS: Windows 10; CPU: Intel Core i5-3210 M; RAM: 8GB) running Java (version 1.8.0_45). In contrast, it took over 180 person-hours to create the gold standard matrix.

Table 3 shows the performance scores of MicroPIE with the Character Predictor (i.e., SVM) enabled. The number of values in the GSM and MicroPIE matrix is shown for each character as well. MicroPIE extracted 4049 total values, resulting in a total relaxed hit score of 3198.5, out of the maximum total values of 4098 from the GSM (Table 3). In other words, MicroPIE extracted 79.0% of character values successfully. Half of the 42 characters had Relaxed_F1 scores greater than 0.80. Among those, the Relaxed_F1 score for 11 characters was greater than 0.90, the Relaxed_F1 score for 16 characters was between 0.50 and 0.80, while the Relaxed_F1 score for only 5 characters was equal to or below 0.50. Of all the characters, 13 had the same rigid and relaxed scores, while the other 29 characters had greater relaxed scores. Thus, MicroPIE extracted at least partially correct values for most characters.

**Table 3:** Performance of MicroPIE with Character Predictor

Character	Extraction methods	# of GSM Values	# of MicroPIE Output Values	P	R	F1	Relaxed_P	Relaxed_R	Relaxed_F1
**%G + C**	linguistic rules ^N^	90	96	0.91	0.97	0.94	0.91	0.97	**0.94**
Cell Shape	term matching ^**S**^	125	166	0.49	0.65	0.56	0.64	0.84	0.73
**Cell Diameter**	linguistic rules ^N^	14	18	0.67	0.86	0.75	0.72	0.93	**0.81**
**Cell Length**	linguistic rules ^N^	68	68	0.89	0.89	0.89	0.93	0.93	**0.93**
**Cell Width**	linguistic rules ^N^	56	58	0.91	0.95	0.93	0.93	0.96	**0.95**
**Cell Relationships & Aggregations**	term matching ^**S**^	25	27	0.72	0.78	0.75	0.82	0.88	**0.85**
**Gram Stain Type**	term matching ^**S**^	64	62	1.00	0.97	0.98	1.00	0.97	**0.98**
External Features	term matching ^**S**^	23	21	0.55	0.50	0.52	0.62	0.57	0.59
**Internal Features**	term matching ^**S**^	63	56	0.78	0.69	0.73	0.91	0.81	**0.86**
**Motility**	term matching ^**S**^	76	77	0.71	0.72	0.71	0.84	0.86	**0.85**
**Pigment Compounds**	term matching ^**S**^	58	51	0.90	0.79	0.84	0.97	0.85	**0.91**
**NaCl Minimum**	linguistic rules ^N^	44	46	0.74	0.77	0.76	0.80	0.84	**0.82**
**NaCl Optimum**	linguistic rules ^N^	33	30	0.92	0.83	0.87	1.00	0.91	**0.95**
**NaCl Maximum**	linguistic rules ^N^	44	46	0.75	0.78	0.77	0.83	0.86	**0.84**
**pH Minimum**	linguistic rules ^N^	24	24	0.92	0.92	0.92	0.92	0.92	**0.92**
**pH Optimum**	linguistic rules ^N^	26	27	0.96	1.00	0.98	0.96	1.00	**0.98**
**pH Maximum**	linguistic rules ^N^	23	24	0.92	0.96	0.94	0.92	0.96	**0.94**
Temperature Minimum	linguistic rules ^N^	58	44	0.89	0.67	0.77	0.89	0.67	0.77
Temperature Optimum	linguistic rules ^N^	62	40	1.00	0.65	0.78	1.00	0.65	0.78
Temperature Maximum	linguistic rules ^N^	58	44	0.91	0.69	0.78	0.91	0.69	0.78
Aerophilicity	term matching ^**S**^	83	89	0.63	0.68	0.65	0.69	0.74	0.72
Magnesium Requirement for Growth	term matching ^**S**^	4	2	0.50	0.25	0.33	1.00	0.50	0.67
Vitamins and Cofactors Used For Growth	term matching ^**S**^	14	26	0.39	0.71	0.50	0.39	0.71	0.50
Salinity Requirement for Growth	linguistic rule + term matching ^**S**^	42	65	0.58	0.89	0.70	0.60	0.93	0.73
**Antibiotic Sensitivity**	linguistic rule + term matching ^**S**^	96	84	0.91	0.80	0.85	0.93	0.81	**0.87**
**Antibiotic Resistant**	linguistic rule + term matching ^**S**^	64	49	0.96	0.73	0.83	0.96	0.73	**0.83**
**Colony Shape**	term matching ^**S**^	102	98	0.97	0.94	0.96	0.98	0.94	**0.96**
**Colony Margin**	term matching ^**S**^	43	44	0.89	0.91	0.90	0.96	0.98	**0.97**
**Colony Texture**	term matching ^**S**^	69	75	0.85	0.92	0.88	0.86	0.94	**0.90**
Colony Color	term matching ^**S**^	80	127	0.53	0.84	0.65	0.59	0.93	0.72
Fermentation Products	linguistic rules + term matching ^**S**^	127	141	0.59	0.66	0.62	0.64	0.71	0.67
Other Metabolic Product	term matching ^**S**^	13	56	0.07	0.31	0.12	0.07	0.31	0.12
Pathogenic	term matching ^**S**^	3	3	0.50	0.50	0.50	0.67	0.67	0.67
Disease Caused	term matching ^**S**^	7	11	0.27	0.43	0.33	0.36	0.57	0.44
Pathogen Target Organ	term matching ^**S**^	4	9	0.22	0.50	0.31	0.22	0.50	0.31
Haemolytic & Haemadsorption Properties	term matching ^**S**^	10	7	0.57	0.40	0.47	0.57	0.40	0.47
Organic Compounds Used Or Hydrolyzed	term matching ^**S**^	620	480	0.85	0.66	0.74	0.89	0.69	0.77
Organic Compounds Not Used Or Not Hydrolyzed	term matching ^**S**^	733	468	0.92	0.58	0.71	0.92	0.59	0.72
Inorganic Substances Used	term matching ^**S**^	36	45	0.59	0.74	0.65	0.61	0.76	0.68
Inorganic Substances Not Used	term matching ^**S**^	61	41	0.81	0.54	0.65	0.81	0.54	0.65
Fermentation Substrates Used	linguistic rules + term matching ^**S**^	411	629	0.57	0.88	0.69	0.59	0.91	0.72
**Fermentation Substrates Not Used**	linguistic rules + term matching ^**S**^	442	475	0.85	0.91	0.88	0.86	0.93	**0.89**
*In total*		*4098*	*4049*	*Total relaxed hit scores*			*3198.5*		

*Abbreviations*: *Superscript N* numerical character, *S* string-based/categorical character. The characters with > = 0.8 in Relaxed_F1 score are shown in bold

### The performance of MicroPIE without character predictor

**Fig. 6:**
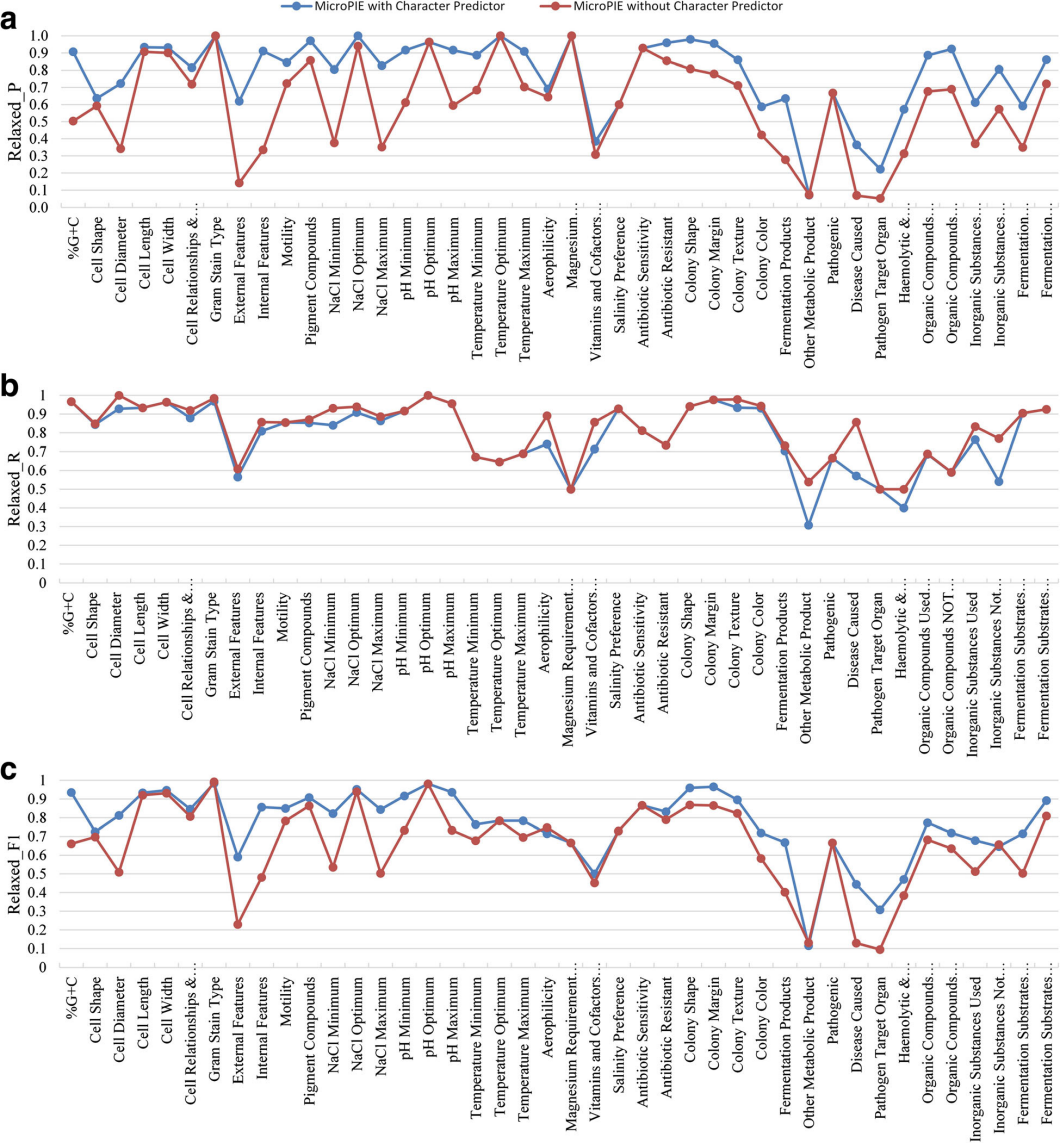
The performance comparison between MicroPIE with and without Character Predictor. **a** Relaxed_P, **b** Relaxed_R, and **c** Relaxed_F1 scores

The performance scores for MicroPIE with the Character Predictor (SVM) component disabled were also calculated. Without Character Predictor component choosing the appropriate character extractor, MicroPIE applied all the character extractors to each sentence and extracted a total of 5969 values, 45.7% more than what was in GSM (4098). Compared to MicroPIE with SVM, the total relaxed hit score was increased by 61.5 (from 3198.5 to 3260). At the same time, about 2000 incorrect values were also extracted, resulting in a decreased accuracy of 54.6% (3260/5969), 30.8% lower than the accuracy of MicroPIE with SVM (79.0%). The performance differences in Relaxed_P, Relaxed_R and Relaxed_F1 scores with and without Character Predictor are presented in Fig. 6. MicroPIE with SVM achieved higher Relaxed_P scores for 34 characters and resulted in a slightly lower score for only 1 character (Other Metabolic Product; Fig. 6a). MicroPIE without SVM achieved higher Relaxed_R scores for 22 characters (Fig. 6b). Note that the differences in recall (Relaxed_R) were smaller than they were in precision (Relaxed_P). The combined effect on MicroPIE performance can be seen in Fig. 6c in terms of Relaxed_F1 scores: MicroPIE with SVM performed better for 32 of the characters but performed worse for 2 characters (Gram Stain Type and Inorganic Substances Not Used), albeit by a small margin (Relaxed_F1 score of 0.008 and 0.010, respectively).

### Comparison between MicroPIE and student performance

The performance scores of the student-generated matrix were compared to those of the MicroPIE-generated matrix. MicroPIE performed significantly better than undergraduate microbiology students (Table 4). The total relaxed hit score of the MicroPIE matrix (445) was 123% higher than that of student-generated matrix (200). In addition, MicroPIE consistently performed better than students on all the compared characters and in all metrics (Table 4). Student's t-Tests on the description-based Relaxed_P, Relaxed_R and Relaxed_F1 scores indicate statistically significant differences between MicroPIE and student performances (*p*-value < 0.01).

**Table 4:** The performance comparison between the students and MicroPIE on 12 characters in 46 taxonomic descriptions

Character	Student Output			MicroPIE Output		
	Relaxed_P	Relaxed_R	Relaxed_F1	Relaxed_P	Relaxed_R	Relaxed_F1
Motility	0.35	0.25	0.29	0.89	0.86	0.87
Pigment Compounds	0.07	0.13	0.09	1.00	0.96	0.98
pH Minimum	0.58	0.75	0.65	0.91	0.91	0.91
pH Optimum	0.72	0.59	0.65	0.96	1.00	0.98
pH Maximum	0.60	0.79	0.68	0.91	0.95	0.93
Temperature Minimum	0.67	0.35	0.46	0.92	0.87	0.90
Temperature Optimum	0.63	0.21	0.31	1.00	0.88	0.94
Temperature Maximum	0.75	0.34	0.47	0.95	0.88	0.91
Aerophilicity	0.52	0.55	0.53	0.71	0.77	0.74
Antibiotic Sensitivity	0.57	0.37	0.45	0.94	0.87	0.90
Antibiotic Resistant	0.91	0.70	0.79	0.98	0.80	0.88
Fermentation Products	0.47	0.28	0.35	0.69	0.69	0.69

## Discussion

### MicroPIE performance analysis

**Fig. 7:**
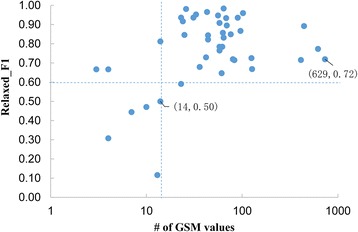
Scatter plot showing the relationship between Relaxed_F1 scores and frequency of character value occurrence in GSM for each of the 42 characters. The axis of the number of GSM values is log-transformed

Characters with more than 14 character values in the GSM produced Relaxed_F1 scores greater than 0.60 (Fig. 7), indicating that MicroPIE performed better when character values appeared more frequently in the GSM. These same characters also appeared more frequently in the corpus of 625 taxonomic descriptions, providing abundant domain terms and robust linguistics rules during the development of MicroPIE and hence resulting in a final algorithm that can more reliably extract the appropriate target values.

The higher performance (Relaxed_F1 score > 0.70) for the 13 numerical characters (superscript N in Table 3) reflects the effectiveness of various linguistic rules. Linguistic rules, specifically syntactic patterns, also resulted in higher performance for some string-valued characters (superscript S in Table 3) when useful patterns about the character values were well captured by the extractors; for example, Antibiotic Sensitivity and Antibiotic Resistant achieved high Relax_F1 scores of 0.87 and 0.83 respectively.

The term matching method achieved high extraction performance for some characters as well, such as Gram Stain Type and Colony Shape (Table 3). However, multiple factors could contribute to the failure of term matching method (e.g., External Features, Disease Caused, and Pathogen Target Organ). The coverage of the term lists may be limited (particularly for character values with low frequencies of occurrence), terms may be included in the lists of multiple characters making it difficult to determine the correct character in a specific sentence, or terms may be only part of a value to be extracted (for example, the term “brown” in the term list for Colony Color is part of the value “slightly brown”). In addition, MicroPIE attempts to extract a complete phrase as opposed to a single word, but this does not solve the cases where one phrase contains multiple values (e.g., Gram Strain Type and Cell Shape expressed in one phrase “Gram-negative rods”) or where one value is expressed across phrase boundaries (e.g., Cell Shape: “rod-shaped with slightly irregular sides and pointed ends”). Learning the relationships within and between phrases will help address these problems.

During the development of the GSM and evaluation of MicroPIE, we experienced a fair amount of tension between the need to extract as much information as possible and the need to fit the values in the matrix meaningfully so the values of each character were comparable between taxa. This largely stemmed from the variations in description content, and sometimes the vagueness of the language, in taxonomic descriptions. For example, the following sentence,

“Growth occurs at −2 °C in marine broth. Good growth occurs on MA at 1–25 °C. No growth occurs at 30 °C or higher”,

is ambiguous with respect to the optimal growth temperature. Although experts could judge “1–25 °C” as the optimal growth temperature (Temperature Optimum, as done in the GSM), MicroPIE identified “1–25 °C” as a temperature related growth condition, but did not output the value as the optimal. Such ambiguity presents a challenge for MicroPIE. Thus, we designed MicroPIE to adhere to the principle of “stay true to the original descriptions” and to not attempt to make a judgement that needs much expert knowledge.

### The effects of character predictor

MicroPIE with the SVM Character Predictor showed significantly higher precision than MicroPIE without Character Predictor (Fig. 6a). With highly accurate character prediction, sentences containing specific characters were triaged to their specific character extractors, rather than to all character extractors non-discriminatorily. The triage alleviated issues in term matching caused by terms that are shared by multiple characters. For example, the term “filamentous” appears in term lists of both Cell Shape and Colony Shape. Thus, MicroPIE without Character Predictor extracted this term as values of both characters, while Character Predictor utilized the information in the complete sentence to predict the correct character so that the correct term list was used. Our results show that using SVM resulted in increased Relaxed_P scores for five characters with shared terms in the term lists: Cell Shape improved by 0.04, External Features 0.48, Internal Features 0.58, Colony Shape 0.17, and Colony Texture 0.15.

Precision and recall scores are known to have an inverse relationship [54]—higher precision often means lower recall—which is also manifested in this case with the slightly lower recalls for MicroPIE with Character Predictor (Fig. 6b). Characters showing the larger decreases in recall scores were the characters with values having low frequencies of occurrence (i.e., Vitamins and Cofactors Used For Growth, Disease Caused, and Haemolytic & Haemadsorption Properties) and hence a few missed values resulted in much lower recall scores. For these characters, the insufficient training examples (<50) affected the SVMs ability to make correct predictions for new data (i.e., the test taxonomic descriptions). Although more training examples were available for NaCl Minimum, Aerophilicity, and Inorganic Substances Not Used, the Character Predictor predicted more than 10% of relevant sentences as irrelevant, resulting in decreased recall performance for these characters. This suggests that the training examples of these characters were not very good representatives of the test descriptions. Despite the lower recall scores, the extraction accuracy and F1 scores suggest that SVMs contributed positively to MicroPIE performance.

### Comparison between MicroPIE and student performance

A detailed analysis of student’s matrix showed that their extraction errors may be attributed to students’ inability to understand the descriptions completely. Their lower performance suggests that, lacking sufficient domain knowledge, novices with only some background knowledge are not capable of extracting microbial characters with high accuracy. The performance comparison between MicroPIE and students highlights the challenges in this task and the need for high quality domain knowledge for computer algorithms to perform well and further improve.

### Differentiating MicroPIE with entity annotators

Many annotation tools (e.g., BioPortal Annotator [55]) match individual phrases in textual descriptions to ontology terms. Such systems are “Entity Recognition” systems, where individual entities (represented by ontology terms) are recognized, but relationships among these entities are not identified. In contrast, a key function of MicroPIE is to relate character values to appropriate characters. Once the character values are extracted and related to their characters, they can then be matched to terms in ontologies using algorithms such as BioPortal Annotator algorithm (see Future Work).

### Future work

Although the performance of current MicroPIE is promising, we have identified several areas it can be further improved. New methods for detecting extraction boundaries and for constructing linguistic rules automatically need to be explored. One such candidate is Unsupervised Semantic Parsing (USP), which combines Markov network and first-order logic to cluster terms with similar semantic roles based on their syntactic dependencies with surrounding text [56]. With this technique, instead of handcrafting a rule to extract anything following the verb “hydrolyze”, the cluster of terms, “hydrolyse”, “hydrolyze” and “degrade”, will enable MicroPIE to generate a more robust rule of extracting anything following any of the terms in the cluster.

Another way in which MicroPIE could be improved would be to replace simple term lists with an ontology. The term lists in this study formed the foundation for a new microbial ontology, MicrO [57]. MicrO contains classes of phenotypic and metabolic characters, assays, and culture media of prokaryotes, connecting terms to 19 other ontologies using logical axioms. It also includes a large number of synonyms and alternate spellings for terms found in the corpus of taxonomic descriptions used in this study. MicrO would provide a richer knowledge base for MicroPIE, and would assist in the disambiguation of competing characters. It would also provide a controlled vocabulary for characters and character states, and identify synonymous phrases that frequently occur in prokaryotic taxonomic descriptions. In addition, the logical axioms in the ontology would make it possible for higher-order knowledge to be automatically inferred from extracted characters (e.g., a particular species is a heterotroph if it metabolizes an organic compound, such as glucose). Future versions of MicroPIE will utilize MicrO and a few other ontologies such as the Cell Ontology (CL [58]) and the Ontology of Units of Measure (OM [59]) to improve extraction performance and also standardize character output with ontology terms. In addition to a lot of new microbial phenotypic terms, MicrO has imported many terms relevant to our task from other ontologies (and at the same time filtered out irrelevant terms), using it in MicroPIE is the most logical way forward.

A larger number of training sentences and expanded knowledge resources (e.g., disease name ontologies, organ name ontologies) will also improve the performance of character extraction methods, especially for those characters with fewer occurrences in the current training sentences and the GSM (Fig. 7). MicroPIE could also be improved by expanding the variety of target characters for extraction. The current version of MicroPIE does not extract characters such as microbial filterability, habitats, physiological tests, lipids, and hosts. MicroPIE also could be enhanced by giving it the capability of discovering new characters from taxonomic descriptions, and by processing descriptions from different microbial taxa, e.g., cyanobacteria and eukaryotic phototrophs which follow the Botanical Code rather than the Bacteriological Code [60].

Although MicroPIE was designed to extract data from semi-structured taxonomic descriptions, in future experiments, we intent to evaluate its performance on other description texts, such as less structured descriptions of prokaryotic taxa in PubMed or in online databases (such as http://www.omicsdi.org/), and to research and expand its capability and robustness. The combination of data extracted from semi-structured descriptions and incorporation of the rich MicrO ontology will certainly be useful for MicroPIE to tackle different description styles/formats.

## Conclusion

We report the development and evaluation of MicroPIE, a phenotypic information extraction system for prokaryotic taxonomic descriptions. We show that MicroPIE greatly increases the speed by which phenotypic character information can be extracted from published taxonomic descriptions. Evaluation against a hand-generated gold standard matrix showed that MicroPIE performed well (Relaxed_F1 score > 0.80) on over half of designated characters and achieved an overall accuracy of 79.0% and overall performance that was significantly better than the performance of undergraduate microbiology students. It was also shown that inclusion of the Character Predictor component using SVM (Support Vector Machine) improved the overall performance of MicroPIE. We therefore conclude that MicroPIE has the potential to significantly accelerate the construction of phenotypic character matrices for prokaryotic taxa, removing a fundamental roadblock for the study of large-scale character evolution in microorganisms. We expect future improvements will make it more robust and effective.

## Abbreviations

G + C: DNA molar guanine and cytosine content
GSM: Gold standard matrix
LSA: Latent semantic analysis
MicroPIE: Microbial Phenomics Information Extractor
NLP: Natural language processing
OIE: Open information extraction
POS: Part-of-speech
SVM: Support vector machine
UTF-8: Universal Transformation Format-8
